# Optogenetic control of mesenchymal cell fate towards precise bone regeneration

**DOI:** 10.7150/thno.36455

**Published:** 2019-10-18

**Authors:** Weicai Wang, Delan Huang, Jianhan Ren, Runze Li, Zhicai Feng, Chenyu Guan, Baicheng Bao, Bin Cai, Junqi Ling, Chen Zhou

**Affiliations:** 1 Guanghua School of Stomatology, Hospital of Stomatology, Guangdong Provincial Key Laboratory of Stomatology, Sun Yat-sen University, 56 Lingyuanxi Road, Guangzhou 510055, China.

**Keywords:** Optogenetics, bone tissue regeneration, gene expression control, proliferation and differentiation

## Abstract

**Rationale:** Spatial-temporal control of cell fate *in vivo* is of great importance for regenerative medicine. Currently, there remain no practical strategies to tune cell-fate spatial-temporally. Optogenetics is a biological technique that widely used to control cell activity in genetically defined neurons in a spatiotemporal-specific manner by light. In this study, optogenetics was repurposed for precise bone tissue regeneration.

**Methods:** Lhx8 and BMP2 genes, which are considered as the master genes for mesenchymal stem cell proliferation and differentiation respectively, were recombined into a customized optogenetic control system. In the system, Lhx8 was constitutively expressed, while BMP2 together with shLhx8 expression was driven by blue light.

**Results:** As expected, blue light induced BMP2 expression and inactivated Lhx8 expression in cells infected with the optogenetic control system. Optogenetic control of BMP2 and Lhx8 expression inversely regulates MSC fate *in vitro*. By animal study, we found that blue light could fine-tune the regeneration *in vivo*. Blue light illumination significantly promotes bone regeneration when the scaffold was loaded with MSCs infected with adeno-Lhx8, GI-Gal4DBD, LOV-VP16, and BMP2-shLhx8.

**Conclusions:** Together, our study revealed that optogenetic control of the master genes for mesenchymal stem cell proliferation and differentiation would be such a candidate strategy for precise regenerative medicine.

## Introduction

Spatial and temporal gene expression is essential for development and regeneration in multicellular systems^1^-^3^. For tissue engineering, spatial and temporal control of gene activation or repression is also needed to recapitulate the heterogeneous complexity and architecture of the tissues intended to model or replace^2^. Synthetic gene regulation systems to mimic the developmental dynamics of target genes are recently intensively studied^4^. Optogenetic systems, which were originally developed for basic neuroscience 5, 6, are becoming unique tools to spatially and temporally control gene expression^7^-^11^. In the system, light-inducible noncovalent protein-protein interactions were included to activate or repress gene expression. As the noncovalent protein-protein interactions are reversible, allowing for dynamic control of gene expression.

Up to now, mul tiple natural protein interactions induced by light have been repurposed for optogenetic control of gene expression. For example, the light-dependent binding of FKF1 to GIGANTEA (GI), have been engineered to develop a technology called light activated dimerization (LAD) to artificially induce protein hetero- and homodimerization in live cells using light^12^. FKF1 and GIGANTEA (GI) are two proteins that control flowering in Arabidopsis thaliana^12^-^15^. FKF1 contains a LOV (light, oxygen or voltage) domain that detects light using flavin mononucleotide (FMN). Illumination with 450 nm blue light induces formation of a covalent bond between FMN and cysteine 91 of FKF1, which then allows FKF1 to bind to the nuclear protein GI. The FKF1-GI interaction could be reversible when cysteinyl-flavin bond is hydrolyzed^16^. It can be engineered to generate a light-activated transcription factor by fusing domains of GI and FKF1 to the DNA binding domain of Gal4 and the transactivation domain of VP16, respectively^12^. Till now, there were few studies of application of optogenetic control system for regenerative medicine.

Lhx8 (LIM Homeobox 8), which is also known as L3 and Lhx7, is a remarkably conserved transcriptional factor of the LIM-homeobox family among species. Lhx8 transcripts were detected abundantly in certain periods of multiple mesenchymal lineages, including dental mesenchyme at bud stage (E12.5) 17-20. It has been well established that Lhx8 plays crucial roles in regulating the cell fates. We have previously revealed that Lhx8 regulates mesenchyme development as a negative gatekeeper via fine-tuning Wnt/β-catenin and TGFβ (transforming growth factor-β) pathways^21^. Like other bone morphogenetic proteins, BMP2 (bone morphogenetic protein-2) plays an important role in the development of bone and cartilage. Recombinant human protein (rhBMP-2) is clinically used for orthopaedic purposes in the United States^22^. Regarding the different function of Lhx8 and BMP2 in bone formation, spatial-temporal regulation of Lhx8 and BMP2 mimicking the developmental dynamics would precisely remodel the bone regeneration.

We here established an optogenetic expression system, which could inactivate Lhx8 expression and activate BMP2 expression simultaneously under the control of blue light. We further showed that prior expression of Lhx8 promotes proliferation of MSC (mesenchymal stem cell), while inactivation of Lhx8 together with induction of BMP2 by blue light significantly promotes osteogesis. Moreover, blue light could fine-tune bone regeneration in the critical size calvarial defect repair model. Our study revealed that optogenetic control of the master genes for mesenchymal stem cell proliferation and differentiation would be such a candidate strategy for precise regenerative medicine.

## Materials and Methods

### Plasmid construction

GI-Gal4DBD, LOV-VP16, 5×Gal4 UAS-GFP-shScramble, 5×Gal4 UAS-BMP2-shscramble, 5×Gal4 UAS-BMP2-shLhx8 were synthesized by Genscript (Nanjing, China). GI-Gal4DBD and LOV-VP16 were then subcloned into the pWPI vector using the PacI restriction sites, allowing transcription of GI-Gal4DBD, LOV-VP16 under the control of EF1α promoter. The synthesized 5×Gal4 UAS-GFP-shScramble, 5×Gal4 UAS-BMP2-shscramble, 5×Gal4 UAS-BMP2-shLhx8 were cloned into the pWPI with Cla1 and BstB1 enzymes respectively, by which the original EF1α promoter in pWPI was replaced with the 5×Gal4 UAS promoter. To over express Lhx8 or the control GFP by adenovirus, the coding sequences of Lhx8 and GFP were cloned into pAdeasy vector respectively as instructed. The Lhx8 used was mouse origin, and BMP2 was human origin, as they are highly conserved to the corresponding homologues in rat. The shLhx8 sequence targets both endogenous and exogenous transcripts. Descriptions of plasmid insertion sequences in this work are given in** Supplementary data**.

### Bone marrow mesenchymal stem cell (MSC) isolation

SD rat (4-6 weeks old) were obtained from the animal center of Sun Yat-sen University. All the procedures were approved by the Institutional Animal Care and Use Committee (IACUC) of Sun Yat-sen University. For bone marrow MSC isolation, the rats were sacrificed by euthanization followed by cervical dissection. Bone marrows of the femur and tibia were harvested by syringe flushing. The cells were pooled, pelleted, resuspended, and cultured as described before^23^, ^24^. Cell cultures at indicated time were further applied for either *in vivo* or *in vitro* experiments in this study.

### Virus packaging and infection

Control pWPI or Lhx8, GI-Gal4DBD, LOV-VP16, 5×Gal4 UAS-BMP2-shLhx8 in pWPI vector (6.25μg), pMD2.G (Plasmid #12259, Addgene) (0.625 μg) and psPAX2 (Plasmid #12260, Addgene) (3.125μg) vectors were co-transfected into 80% confluent HEK293T cells using Calcium Phosphate Transfection Kit per manufacturer's protocol (Invitrogen). The supernatant containing corresponding virus was collected 2 days after transfection and then filtered with 0.45 μm membrane, followed by purification with ViraBind™ Lentivirus Purification Kit (Cell Biolabs) per manufacturer's instructions. MSCs were cultured to 30-50% confluence and infected with lentivirus in 8 μg/mL polybrene (Santa Cruz Biotechnology). For adenovirus mediated Lhx8 expression, the CDS of Lhx8 was cloned into pAdeasy vector and packaged as instructed. Adenovirus expressing GFP served as a control. Infected cells were analyzed and confirmed by FACS based on GFP expression and were further passaged 3-5 times for light induction or directly used for *in vivo* regeneration.

### Cell transfection and Luciferase reporter assay

The human HEK293 cells were originally purchased from ATCC and cultured in DMEM medium supplemented with 10% fetal bovine serum and 1% penicillin/streptomycin at 37°C containing 5% CO_2_. Cells were passaged at the confluence of 90%. HEK293 cells seeded in the 24 well plate were pretreated with serum free medium for 6 hours and then transfected with indicated plasmids (GI-Gal4DBD, LOV-VP16, 5×Gal4 UAS-Luc) and 50 ng internal control pRL-TK using Lipofectamine 2000 (Invitrogen, Carlsbad, CA) according to the manufacturer's protocol. The medium was changed to culture medium 6 hours later. Then the cells were kept in dark for 12 hours before light administration. After light induction for indicated periods, cells were harvested by passive lysis buffer and subjected to relative luciferase activity assay as instructed by the protocol.

### qPCR

Total RNA was isolated from cells with indicated treatments using the TRizol (Invitrogen), according to the manufacturer's protocol. Genomic DNA contamination was removed by DNase treatment for 30 min at room temperature, followed by DNase inactivation and RNA reconcentration. Reverse transcription was done using the M-MLV kit (Promega) under the manual instruction. Quantitative PCR were run in triplicate using the ABI7500 system. Mean cycle threshold (Ct) values for both BMP2 and Lhx8 were quantified and normalized to Gapdh expression. Relative expression was calculated with the 2^-dd^Ct method. To detect both endogenous and exogenous gene expression, primers common for Lhx8 or BMP2 across different origins were designed. The primers used were listed in **Supplementary Table 1**.

### Light stimulation

To conduct light stimulation on the cultured cells, cells were first seeded on individual plates for easy manipulation. Light stimulation was conducted 48 hours post transfection. Briefly, blue light (450 nm, at the dose of 0.1 mW/cm^2^, 0.5 mW/cm^2^, 1.0 mW/cm^2^, 1.5 mW/cm^2^, 2.0 mW/cm^2^) was administered to the cells by a custom LED light source for indicated periods. Nontreated samples were kept in the dark for the duration. For the *in vivo* experiment, the transplantation sites were covered with band-aid when the light was not conducted to keep the transplantation in dark. For light induction, the band-aid was removed and the customised LED lamp (450 nm, 300 mW) was placed 1 meter atop of the cage. The rats were illuminated with blue light twice a day and 30 min each time.

### EdU Staining

To label the S-phase cells, EdU (5-ethynyl-20-deoxyuridine) incorporation assay was included. Briefly, MSCs were cultured on glass coverslips and incubated with 10 μM EdU for 1 hour, washed, fixed in 3.7% PFA for 15 minutes, permeabilized with 0.1% Triton X-100 for 15 minutes. Then, the cells were reacted with Click-iT Alexa-594 dye-conjugate cocktail for 30 minutes. Samples were counterstained with Hoechest 33342 to visualize nuclei. Images were acquired using a LSM780 confocal microscope (Zeiss, Germany).

### Osteogenic induction and Alizarin Red staining

For osteogenic differentiation, 70% confluent cells were induced by osteogenic induction medium, which contains 10 mM disodium β-glycerophosphate, 0.1 μM dexamethasone, and 50 mg/mL L-ascorbic acid. The osteogenic medium was changed every 3 days. Osteogenic differentiation was examined by Alizarin Red S staining as described previously 21.

For quantification of the osteogenesis, destaining was conducted by adding a 10% cetylpyridinium chloride solution after Alizarin Red staining. Absorbance was measured in a 96-well plate reader at 562 nm.

### CCK-8

For cell proliferation analysis, two thousand cells in 150 μL medium were seeded per well in 96-well plate and received indicated treatments. At the end of the treatments, 10 μL CCK-8 reagents were gently added and mixed evenly into each well. After 2 hours of incubation at 37∘C, the absorbance value was read at 450 nm.

### PLGA scaffold construction and cell loading

Poly(lactic-co-glycolic acid) (PLGA) copolymer scaffolds were fabricated as described before^25^-^27^, with minor modifications. Briefly, 5 g 75:25 PLGA (Sigma-Aldrich) was dissolved in 90 mL dichloromethane (DCM). Then, the PLGA/DCM solution was poured into the glass dish, which was covered by 20 g salt particles with the diameter varying from 125 μm to 300 μm earlier. Then, another 25 g NaCl salt particles were added, as soon as the PLGA/DCM solution sank down to NaCl particles. All the procedures were done in the chemical hood and the DCM was removed by evaporation. NaCl was washed by flushing with large amounts of water. PLGA discs with diameter of 5 mm were obtained via punch. The obtained discs were sterilized by UV irradiation and further immersed into 50 mg/mL collagen I solution for one hour to increase the compatibility with cells. The MSCs-scaffold complex was manipulated by dropping 40 μL MSCs solution (about 1 million cells in number) onto the scaffold. The acquired implants were additionally cultured in the osteogenic medium for 24 hours before being transplanted *in vivo*.

### Critical size calvarial defect model and MSCs-scaffold transplantation

Adult male SD rats (250-300 g in weight), were included for critical size calvarial defect repair. Before surgery, the surgical field was shaved and sterilized. The rats were intraperitoneally injected with pentobarbital (1%, 0.4 mL/100 g) and then subjected to local anesthesia with primacaine. About 2 cm long incisions were made approximately from the occipital middle region to the nose, and an about 5mm full-thickness bone defects were created as described before^28^. The rats were then receiving different implants as indicated. Following suture, postoperative anti-inflammation treatments with gentamycin were conducted. The animals were then fed as before and subjected to light stimulation as described above. All animal procedures were performed in accordance to the Care and Use of Laboratory Animals of Sun Yat-Sen University and were approved by Institutional Animal Care and Use Committee (IACUC) of Sun Yat-Sen University (Approval number: SYSU-IACUC-2018-000041).

### microCT examination

microCT examination was done as described before^28^. After euthanasia, the calvariae around the bone defect were excised and placed in 10% neutral buffered formaldehyde for 24 hours. High-resolution micro-CT scanner (Scanco Medical μCT 50, Switzerland) was employed for imaging. Samples were scanned with a resolution of 20 μm; afterwards, whole cranium was reconstructed using data analysis software. Five mm in diameter along the edge of defect was set as the region of interest. The bone volume fraction (BVF) was calculated.

### Statistical analysis

All experiments were performed with a minimum of three independent biological replicates. Data are expressed as mean ± SEM otherwise indicated. Statistical analysis was performed with GraphPad Prism, using Student's two-tailed t-test for two-group comparison, ANOVA with Tukey's post-hoc analysis for multiple group comparison. Significance was considered when *P*
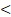
0.05.

## Results

### Optogenetic system for light control of BMP2 and Lhx8 expression

Previous studies have revealed that GI-Gal4DBD and LOV-VP16 could activate gene expression upon blue light. To further optimize the light stimulation dose and duration, the pG5luc vector, in which the luciferase reporter gene was under 5×Gal4 UAS, was included **(Supplementary Figure S1A)**. HEK293 cells transfected with GI-Gal4DBD, LOV-VP16, pG5luc and pRL-TK, were treated with or without 30 min blue light (0-2 mW/cm2) before harvested for luciferase activity. As expected, blue light activated the luciferase expression in a power dependent manner, while 1 mW/cm2 and 2 mW/cm2 treatment had similar effects, suggesting that 1 mW/cm2 might be a reasonable dose in the balance of efficacy and potential toxicity **(Supplementary Figure S1B)**. Among the tested groups, twice 30 min illumination interspaced with 5.5 h activated the gene expression much more significant than other groups **(Supplementary Figure S1C**, **S1D)**. Moreover, when the light illumination was removed, the luciferase activity decreased gradually with time **(Supplementary Figure S1E)**, suggesting that the optogenetic control is reversible.

**Figure 1:**
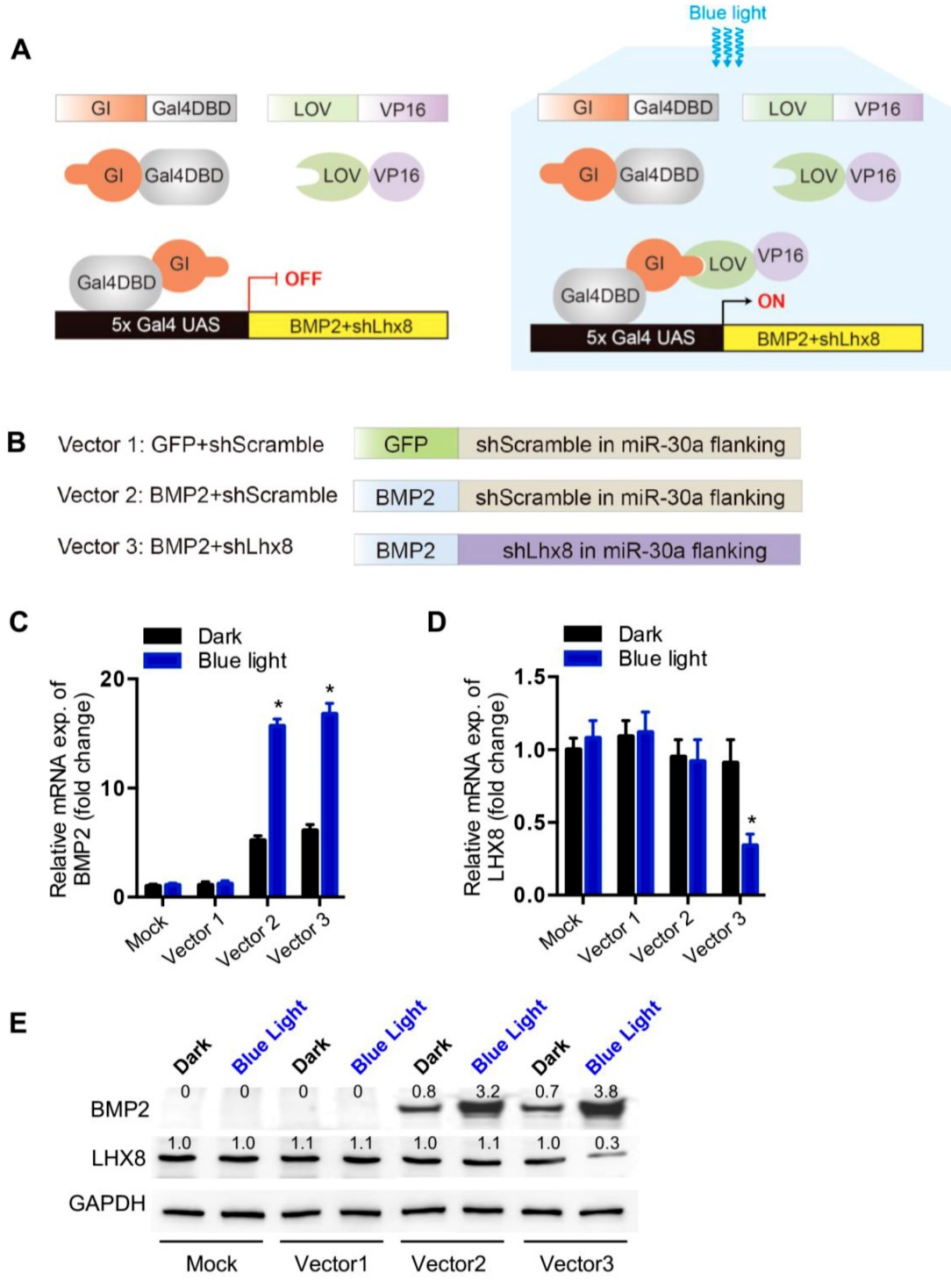
**Optogenetic system for light control of BMP2 and Lhx8 expression. (A)** Schematic representation of the structure of GI-Gal4DBD and LOV-VP16, and their interaction mediated transactivation of the target gene under 5×Gal4 UAS promoter upon blue light. **(B)** Schematic representation of the structure of the transcript encoding GFP, BMP2 and/or shRNA against Lhx8, in which the miR-30a flanking sequence was cloned downstream to the BMP2 stop codon and shLhx8 was inserted into the region where miR-30a localized. The sequences were cloned into pWPI backbone using Cla1 and BstB1 sites together with the 5×Gal4 UAS promoter. **(C-D)** qPCR analysis of the BMP2 expression (C) and Lhx8 (D) in MSC cells with or without blue light illumination. MSCs were infected with BMP2-shLhx8 expressing virus or corresponding controls together with GI-Gal4DBD, LOV-VP16, and cells were treated with or without 30 min blue light (1 mW/cm^2^) 24 hours later, followed by RNA expression analysis. Data were expressed as mean±SEM, * P <0.05. n=5. (E) Western blot analysis of BMP2 and LHX8 expression in MSCs treated same as above. GAPDH served as a loading control. Data were representative of 3 independent experiments. Vector 1: GFP+shScramble; Vector 2: BMP+shScamble; Vector 3: BMP2+shLhx8.

To control mesenchymal stem cell fate with the above optogenetic expression system, especially proliferation versus differentiation, Lhx8 and BMP2 genes were included for their potent function in regulating proliferation and differentiation respectively^21^, ^22^. Briefly, the cells were infected with lentiviruses expressing GI-Gal4DBD, LOV-VP16 driven by EF1α in pWPI, and BMP2-shLhx8 driven by 5×Gal4 UAS **(Figure 1A)**. Theoretically, illumination of blue light would drive the expression of BMP2-shLhx8 **(Figure 1A)**. Previously, miR-30a flanking sequence was widely used to produce shRNA by PolII promoter in a similar manner as miRNAs^29^, ^30^. In the 5×Gal4 UAS-BMP2-shLhx8, the miR-30a flanking sequence was cloned downstream to the BMP2 stop codon and shLhx8 was inserted into the region where miR-30a localized. To this end, the transcript BMP2-shLhx8 could be simultaneously translated to BMP2 protein and processed to shLhx8 **(Figure 1B)**. To test whether the virus could infect MSCs efficiently, MSCs were infected adenovirus or lentivirus expressing GFP. FACS analysis revealed that MSCs could be efficiently infected by either lentivirus or adenovirus **(Supplementary Figure 2)**. Thus, GI-Gal4DBD, LOV-VP16, and 5×Gal4 UAS-BMP2-shLhx8 were packaged in lentivirus system to infect MSCs. As expected, blue light treatment (1 mW/cm2, twice, 30 min each time) significantly induced BMP2 expression in the infected MSCs **(Figure 1C)**, compared with the mock and dark control. Notably, there was a leaky of BMP2 in dark (**Figure 1C**). Simultaneously, the endogenous expression of Lhx8 was significantly repressed by blue light **(Figure 1D)**, with the mean -ΔCt value relative to the internal control Gapdh changed from -2.65 to -4.02. Western blot analysis further confirmed the optogenetic control of BMP2 and LHX8 expression in MSCs by the system **(Figure 1E)**.

### Optogenetic control of BMP2 and Lhx8 expression inversely regulates MSC fates *in vitro*

**Figure 2:**
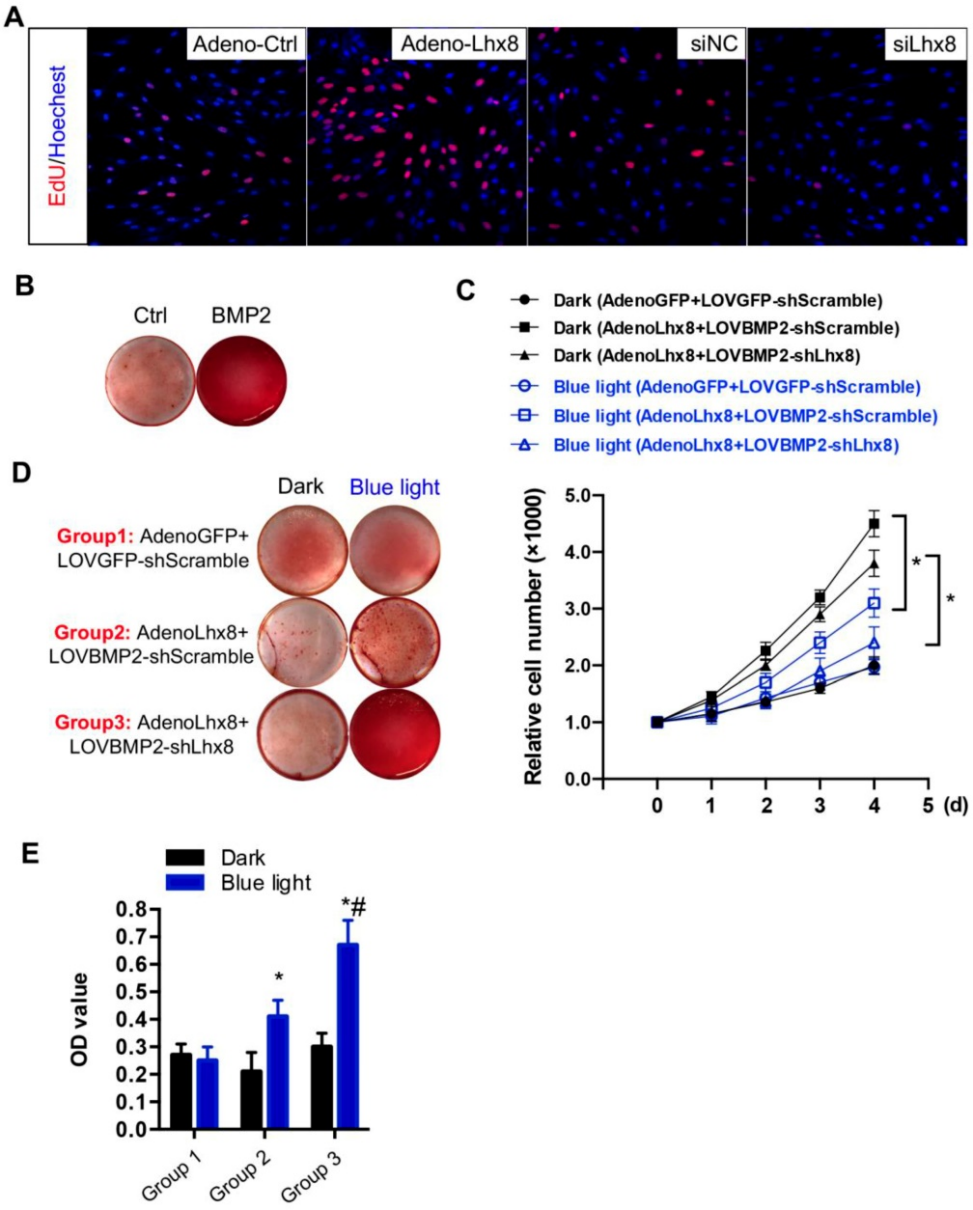
**Optogenetic control of BMP2 and Lhx8 expression inversely regulates MSC fates *in vitro*. (A)** Representative images of the EdU staining (Red) of the cells with Lhx8 overexpressed or knocked down. Nuclei were counterstained with Hoechst. **(B)** Representative images of the Alizarin red S staining of the cells treated with Vehicle or 100 ng/ml BMP2. Cells were cultured in osteogenic medium with or without BMP2 for 14 days. **(C)** Growth curve of MSC cells with indicated treatments. MSCs were infected with GI-Gal4DBD, LOV-VP16, adeno-Lhx8, and BMP2-shLhx8 or corresponding controls. Cells were subjected to dark or blue light (30 min, 1 mW/cm^2^). Cell numbers were counted by CCK-8 assay. Data were expressed as mean±SEM, *P<0.05 by ANOVA. n=5. **(D)** Representative images of the Alizarin red S staining of the cells treated similar as above, except that the cells were cultured in osteogenic medium. Alizarin red S staining was performed after 14-day culture. **(E)** Quantification data of the Alizarin red S staining in Figure 2D. *P<0.05 vs dark; #* P*<0.05 vs blue light in Group 1 and 2. n=5.

Consistent with previous findings^21^, we here also found that Lhx8 promoted MSC cell proliferation as seen from the increased EdU staining cells. In addition, knockdown of Lhx8 in the mesenchymal stem cells significantly inhibited cell growth (Figure 2A). In contrast, BMP2 treatment promoted osteogenesis as expected, as seen from the increased Alizarin S red staining (Figure 2B). Next, we explored whether blue light illumination could switch off the proliferation while switch on the osteogenesis in the MSCs infected with adeno-Lhx8, GI-Gal4DBD, LOV-VP16, and BMP2-shLhx8. Illumination of blue light (30 min, 1 mW/cm^2^) in control cells without the optogenetic expression system had minimal effects on cell proliferation and differentiation (Supplementary Figure 3A, 3B). In contrast, blue light induced BMP2 significantly inhibited the cell growth, as seen from the differences between blue light and dark in cells treated with AdenoLhx8+LOVBMP2-shScramble (Figure 2C). Additional knockdown of Lhx8 by blue light induced shLhx8 expression further inhibited the cell growth, as seen from the differences between blue light and dark in cells treated with AdenoLhx8+LOVBMP2-shLhx8 (Figure 2C). Consistent with the inhibited proliferation, blue light induced BMP2 and shLhx8 significantly promoted osteogenesis (Figure 2D-E).

### Optogenetic control of BMP2 and Lhx8 expression promotes bone regeneration *in vivo*

**Figure 3:**
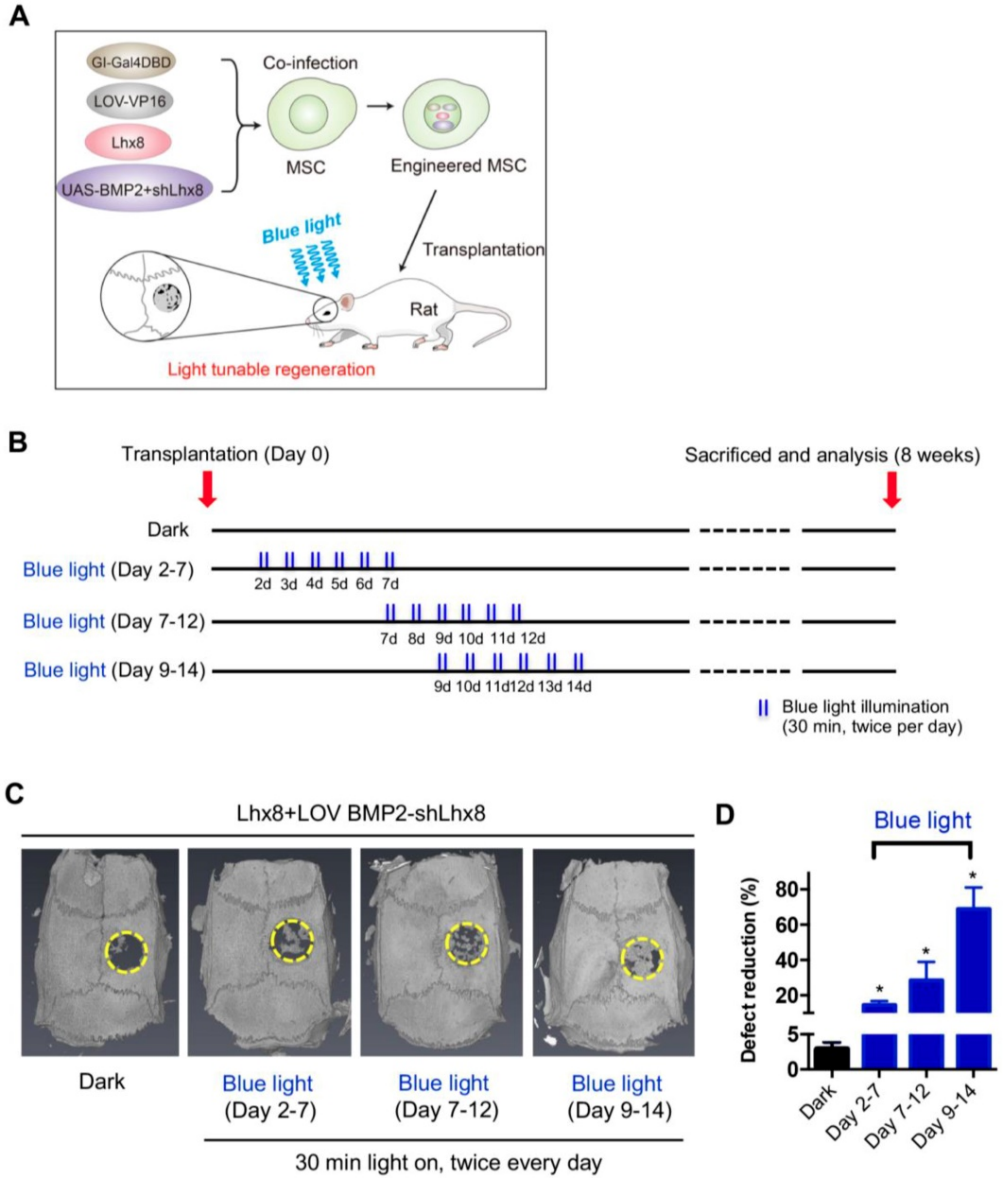
** Optogenetic control of BMP2 and Lhx8 expression promotes bone regeneration *in vivo*. (A)** Scheme of the experimental procedure. MSCs were infected with adeno-Lhx8, GI-Gal4DBD, LOV-VP16, and BMP2-shLhx8. Afterwards, the cells were loaded on the PLGA scaffolds, and the scaffolds were transplanted to the bone defect area. Light was illuminated to promote bone regeneration. **(B)** Schematic representation of the experimental procedure and grouping. **(C)** Representative images showing bone defect healing after 8 weeks of scaffold transplantation. The scaffold was loaded with MSCs infected with adeno-Lhx8, GI-Gal4DBD, LOV-VP16, and BMP2-shLhx8. The rats of each group were subjected to blue light illumination at indicated periods. **(D)** Statistical analysis of the defect reduction in each group. Data were expressed as mean ± SEM, n=5, and *P<0.05.

**Figure 4:**
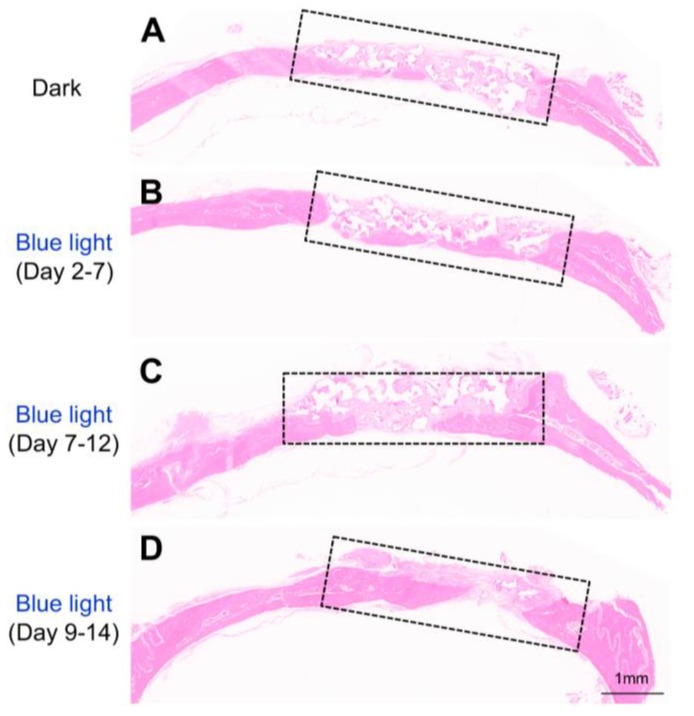
** Histology analysis of the bone regeneration *in vivo*.** Representative HE staining showing bone defect healing after 8 weeks of scaffold transplantation in each group (n=5). The scaffold was loaded with MSCs infected with adeno-Lhx8, GI-Gal4DBD, LOV-VP16, and BMP2-shLhx8. The rats of each group were subjected to blue light illumination at indicated periods and were all sacrificed 8 weeks after transplantation.

In view of above data, we hypothesized that MSCs infected with adeno-Lhx8, GI-Gal4DBD, LOV-VP16, and BMP2-shLhx8 would be beneficial for bone regeneration as the cell fate could be tuned via illumination of blue light. PLGA scaffold was selected for its biodegradability and biosafety. Accordingly, there were about 25% mass degradation after weeks culture in PBS, which was used to mimic the biological fluid though not perfect (Supplementary Figure 4A). In addition, there was no obvious cell toxicity found when MSCs were cultured on the PLGA sheets (Supplementary Figure 4B). Next, the MSCs either with adeno-Lhx8, GI-Gal4DBD, LOV-VP16, and BMP2-shLhx8 infection or corresponding controls were loaded into the PLGA scaffolds (Figure 3A). After the MSCs successfully attached to the PLGA scaffold (Supplementary Figure 5A, 5B), the scaffold was transplanted into the defect area (Supplementary Figure 5C-F, Figure 3A-B). Cells on the scaffold then received illumination, which was performed by a customized LED lamp placed atop of the rat cage. Consistent with the *in vitro* data, blue light illumination had little effects on bone regeneration *in vivo* when the scaffold was loaded with MSCs without any infection (Supplementary Figure 6). In contrast, blue light illumination significantly promoted bone regeneration when the scaffold was loaded with MSCs infected with adeno-Lhx8, GI-Gal4DBD, LOV-VP16, and BMP2-shLhx8 (Figure 3C-D). Notably, compared with other time points, illumination at Day 9-14 resulted in the best regeneration effects (Figure 3C-D). Consistent with the microCT data, histology analysis further confirmed the results (Figure 4). All of these data suggest that promoting proliferation at early stages while differentiation at late stages would be beneficial for bone regeneration, and the proposed light-inducible system are attractive for such a purpose.

## Discussion

In this study, we engineered a light inducible genetic “on/off” switch that can simultaneously induce and inactivate two functional opposite genes via illumination of blue light. The ability to spatial temporal control of gene expression is essential for regenerative medicine. The canonical genetic switches, such as the tetracycline-inducible system and ER-Cre system, which activate or inactivate gene expression via addition of a small molecule or hormone, have been widely used for molecular function study^31^. However, the side-effects limit their application in regenerative medicine^31^. Control release of nanoparticles in the scaffold has also been developed^32^-^34^, but these have relatively poor temporal and spatial resolution and suffer from a lack of specificity. Light-inducible systems are attractive for applications in which high spatial and temporal specificity is needed. It is accepted that the light control system will allow the control of gene expression at the single-cell level in mice^35^. Moreover, they are less likely to cause non-specific effects. Several optogenetic technologies have been developed recently that use light-sensitive proteins to control gene expression^8^, ^9^, ^12^, ^36^. However, it is not easy to regulate multiple genes simultaneously in one system, especially when activation of one gene and inactivation of another simultaneously are needed. Here, we for the first time showed that blue light could tune the Lhx8 and BMP2 dynamic towards efficient regeneration. The cells infected adeno-Lhx8 constitutively express Lhx8 without light, while light inducible BMP2+shLhx8 inactivate Lhx8 and induce BMP2. *In vitro* study revealed that the system could be used to tightly control of cell proliferation and differentiation. We also showed that the optogenetic on/off switch is functional *in vivo*. As to the detailed mechanisms how MSC promotes regeneration *in vivo*, we prefer the idea that timed proliferation and differentiation of MSCs allow enough cells involved in bone regeneration. In addition, during the proliferation, the paracrine signals should be also involved. Since balance of proliferation and differentiation is the dogma of development, the optogenetic control of the balance of genes associated with proliferation and differentiation will enable many applications in basic science and tissue engineering.

Although using an optogenetic tool for the purpose of bone regeneration might be superfluous, it shoud be noted that the importance of the study is that it implicates the optogenetic tool could be applicable for regeneration needs precise spatial/temporal control. The bone regeneration model used here is an easy example to show feasibility of the system. The proposed light tuned regeneration system should be useful for precise regeneration. It is also important to note that there is still a broad scope for improvements, including improving the light dose and timing for better kinetics of gene expression, selection of better gene candidates toward better safety and efficacy, refinement of the FKF1 and GI fragments or application of other light inducible interactions. In addition, light inducible system itself has some other instinct flaws, such as the interference of the sunlight, low penetrance, and toxicity of long time exposure^37^. Another concern about the limitation is the genetic modification of the system. The potential inflammation and immunogenicity caused by optogenetic tools should be strictly evaluated before further application. The safety of the strategy should be the first concern, and it can only be practical before the benefit and potential inflammation and immunogenicity caused by optogenetic tools were balanced, and the possible tumorigenesis derived from differentiation of resistance MSCs were totally excluded. Although the cells are destined to differentiate into osteoblast, migrated and undifferentiated cells should not be a problem. This is a common problem for all the studies based on stem cells. All of these should be kept in mind before future clinical application of the optogenetic system for regenerative medicine. Extension and expansion of the transient light inducible effects via synthetic biology strategy, for example, including a feedback system, might be a solution^8^. Despite of these limitations and flaws, precise control of regeneration by light should be applicable in the near future.
