# Supplementary material for: Optogenetic control of mesenchymal cell fate towards precise bone regeneration
Source: Theranostics. 2019 Oct 18;9(26):8196–205. doi: 10.7150/thno.36455 (PMC6857041; doi:10.7150/thno.36455)
Supplement: Supplementary file 1 — Supplementary figures and tables. [file thnov09p8196s1.pdf]

Supporting Information

Sequences used in the study

GI-GAL4DBD

TTAATTAACCATGGCTAGTTCATCTTCATCTGAGAGATGGATCGATGGTCTTCAGTTCTCTTCCTTG
TTATGGCCTCCGCCACGAGATCCTCAACAACATAAGGATCAAGTCGTTGCTTATGTTGAATATTTT
GGTCAATTTACATCAGAGCAATTCCCAGATGACATTGCTGAGTTGGTCCGGCATCAGTATCCATCA
ACCGAGAAGCGACTTTTGGACGATGTGCTGGCGATGTTTGTCCTTCATCATCCGGAGCATGGTCAT
GCAGTCATTCTTCCAATCATTTCATGTCTTATTGATGGCTCGTTGGTGTACAGCAAGGAAGCTCATC
CGTTTGCCTCTTTCATATCTTTAGTTTGCCCAAGTAGTGAGAATGACTATTCGGAGCAATGGGCTTT
GGCATGTGGAGAAATCCTTCGCATTTTGACTCATTACAACCGTCCCATTTATAAAACTGAGCAGCA
AAATGGAGATACAGAGAGAAATTGTCTGAGCAAAGCTACAACTAGTGGTTCTCCGACTTCAGAGC
CTAAGGCTGGATCACCAACACAGCATGAAAGGAAACCTTTAAGGCCTTTGTCTCCATGGATCAGTG
ATATACTACTTGCTGCTCCTCTTGGTATAAGAAGTGACTATTTCCGATGGTGTAGTGGTGTAATGG
GTAAATATGCTGCTGGAGAGCTCAAGCCGCCAACCATTGCTTCTCGAGGATCTGGTAAACATCCTC
AACTGATGCCTTCAACCCCAAGATGGGCTGTTGCTAATGGAGCTGGTGTCATACTGAGTGTTTGTG
ATGATGAAGTTGCTCGATATGAGACTGCTACGCTGACAGCGGTCGCTGTCCCTGCACTTCTTCTTCC
TCCGCCAACGACATCCTTAGATGAGCATCTAGTTGCTGGCCTTCCAGCTCTTGAACCATATGCACG
TTTGTTTCATAGATACTATGCCATTGCAACTCCAAGTGCTACGCAGAGACTTCTTCTTGGACTCTTA
GAAGCACCACCGTCGTGGGCTCCAGATGCACTTGATGCTGCTGTACAGCTTGTGGAACTCCTTCGA
GCTGCTGAAGATTATGCATCTGGTGTAAGGCTACCCAGGAACTGGATGCATTTGCACTTCTTGCGG
GCTATAGGAATTGCTATGTCTATGAGGGCAGGTGTTGCTGCTGATGCTGCAGCCGCTTTGCTTTTCC
GCATACTCTCACAGCCGGCACTGCTTTTTCCTCCGCTAAGTCAAGTTGAGGGAGTAGAAATTCAGC
ACGCGCCTATTGGTGGCTACAGTTCAAATTACAGAAAACAGATAGAAGTTCCTGCAGCAGAAGCA
ACCATTGAAGCCACTGCCCAAGGAATTGCCTCAATGCTTTGTGCTCATGGTCCTGAAGTTGAGTGG
AGAATTTGCACTATATGGGAAGCTGCTTATGGTTTGATCCCTTTAAATTCTTCGGCGGTTGATCTTC
CCGAAATCATAGTTGCTACCCCACTGCAACCTCCTATCTTGTCATGGAATTTATACATTCCACTCCT
CAAAGTACTTGAATATCTTCCACGGGGGAGTCCTTCGGAAGCATGCTTGATGAAAATATTTGTTGC
CACTGTGGAAACAATACTCAGTAGAACTTTTCCGCCTGAATCTTCCAGGGAACTAACCAGAAAAG
CTAGATCGAGTTTTACCACAAGATCAGCGACCAAAAATCTTGCTATGTCTGAGCTTCGTGCTATGG
TCCATGCTCTCTTTTTAGAATCATGCGCTGGTGTGGAATTAGCTTCACGCCTACTTTTTGTTGTGTTG
ACTGTATGTGTTAGCCATGAAGCACAGTCTAGTGGTAGCAAGAGACCGAGAAGTGAATATGCTAG
TACTACTGAAAATATTGAGGCGAATCAACCTGTATCTAACAATCAAACTGCTAACCGTAAAAGTA
GGAATGTCAAGGGACAGGGACCTGTGGCAGCATTTGATTCATACGTTCTTGCTGCTGTTTGTGCTC
TTGCCTGTGAGGTTCAGCTGTATCCTATGATCTCTGGTGGGGGAACTTTTCCAATTCTGCCGTGGC
TGGAACTATTACAAAGCCTGTAAAGATAAATGGGTCATCTAAAGAGTATGGAGCTGGGATTGACT
CGGCAATTAGTCATACGCGCCGAATTTTGGCAATCCTAGAGGCACTCTTTTCATTAAAACCATCTT

CTGTGGGGACTCCATGGAGTTACAGTTCTAGTGAGATAGTTGCTGCGGCCATGGTTGCAGCTCATA
TTTCCGAACTGTTCAGACGTTCAAAGGCCTTGACGCATGCATTGTCTGGGTTGATGAGATGTAAGT
GGGATAAGGAAATTCATAAAAGAGCATCATCATTATATAACCTCATAGATGTTCACAGCAAAGTT
GTTGCCTCCATTGTTGACAAAGCTGAACCCTTGGAAGCCTACCTTAAGAATACACCGGTTCAGAAG
GATTCTGTGACCTGTTTAAACTGGAAACAAGAGAACACATGTGCAAGCACCACATGCTTTGATACA
GCGGTGACATCCGCCTCAAGGACTGAAATGAATCCAAGAGGAAACCATAAGTATGCTAGACATTC
AGATGAAGGCTCAGGAAGACCCTCAGAGAAGGGTATCAAAGATTTCCTCTTGGATGCTTCTGATCT
AGCGAATTTCCTCACAGCTGATAGACTCGCAGGGTTCTATTGTGGTACACAAAAGCTTTTGAGGTC
AGTGCTTGCAGAGAAACCGGAGCTGTCTTTCTCCGTTGTTTCACTGTTATGGCACAAACTGATTGCT
GCTCCTGAAATCCAGCCCACCGCAGAAAGCACCTCTGCGCAACAAGGATGGAGACAGGTTGTTGA
TGCGCTATGCAATGTCGTATCTGCAACGCCAGCGAAAGCAGCAGCAGCAGTTGTCCTTCAGGCTGA
AAGGGAGTTGCAGCCTTGGATCGCCAAAGATGATGAAGAAGGCCAAAAAATGTGGAAAATCAAC
CAACGGATAGTCAAAGTGTTGGTGGAACTCATGCGCAATCATGACAGGCCTGAGTCACTGGTGAT
TCTCGCAAGTGCATCAGATCTTCTTCTGCGGGCAACTGATGGAATGCTTGTTGATGGAGAAGCTTG
TACATTACCTCAACTTGAGCTACTTGAAGCCACGGCAAGAGCAATACAGCCGGTGCTAGCTTGGG
GGCCATCTGGACTAGCAGTGGTCGACGGTTTATCCAATCTATTGAAGTGTCGTCTACCAGCAACAA
TACGGTGCCTTTCACACCCAAGTGCACACGTACGTGCCTTAAGCACGTCAGTACTACGTGATATCA
TGAACCAAAGCTCCATACCCATCAAAGTAACTCCAAAACTGCCAACAACAGAGAAGAACGGAATG
AATAGTCCGTCCTATCGATTCTTCAACGCCGCCTCAATAGACTGGAAAGCCGATATCCAAAACTGT
TTAAACTGGGAAGCTCACAGCTTGCTCTCCACAACTATGCCTACTCAGTTTCTCGACACTGCGGCT
CGGGAACTCGGCTGTACTATATCCTTGTCCCAAGCGGCCGCAAGTGCTGGTAAGCTACTGTCTTCT
ATCGAACAAGCATGCGATATTTGCCGACTTAAAAAGCTCAAGTGCTCCAAAGAAAAACCGAAGTG
CGCCAAGTGTCTGAAGAACAACTGGGAGTGTCGCTACTCTCCCAAAACCAAAAGGTCTCCGCTGA
CTAGGGCACATCTGACAGAAGTGGAATCAAGGCTAGAAAGACTGGAACAGCTATTTCTACTGATT
TTTCCTCGAGAAGACCTTGACATGATTTTGAAAATGGATTCTTTACAGGATATAAAAGCATTGTTA
ACAGGATTATTTGTACAAGATAATGTGAATAAAGATGCCGTCACAGATAGATTGGCTTCAGTGGA
GACTGATATGCCTCTAACATTGAGACAGCATAGAATAAGTGCGACATCATCATCGGAAGAGAGTA
GTAACAAAGGTCAAAGACAGTTGACTGTATCGCCGTAATTAATTAA

LOV-VP16
ACCATGGCTTACCCATACGATGTTCCAGATTACGCTGCGAGAGAACATGCGATCGGAGAAGCCAC
CGGAAAACGAAAGAAACGCGGCAGAGTTGAAGAAGCAGAAGAATACTGTAACGATGGAATCGAA
GAACAAGTAGAGGATGAGAAGCTTCCGTTAGAGGTTGGGATGTTCTATTACCCAATGACTCCGCCT
TCGTTCATTGTTTCCGATGCTCTGGAGCCAGATTTTCCTTTGATCTATGTCAACAGAGTCTTCGAAG
TCTTCACTGGCTATCGTGCCGATGAAGTTCTTGGTCGTAACTGTCGATTCCTACAGTACAGAGATCC
TCGAGCTCAAAGGCGTCACCCATTGGTTGATCCTGTGGTTGTATCTGAGATTAGGAGATGTCTTGA
AGAAGGTATTGAATTCCAAGACGAGCTTCTTAATTTCAGAAAAGATGGTACTCCTTTGGTTAACAG
ACTACGGCTTGCTCCAATACGTGACGATGATGGAACCATTACACACGTAATTGGGATACAGGTCTT
CTCTGAAACGGGTACCGAAAGCGGCGGGCCGGCCGACGCCCTTGACGATTTTGACTTAGACATGC

TCCCAGCCGATGCCCTTGACGACTTTGACCTTGATATGCTGCCTGCTGACGCTCTTGACGATTTTGA
CCTTGACATGCTCCCCGGGTAA

5×Gal4 UAS-BMP2

ATCGATCGGAGTACTGTCCTCCGAGCGGAGTACTGTCCTCCGACTCGAGCGGAGTACTGTCCTCCG
ATCGGAGTACTGTCCTCCGCGAATTCCGGAGTACTGTCCTCCGAAGACGCTAGCGGGGGGCTATAA
AAGGGGGTGGGGGCGTTCGTCCTCACTCTAGATCTGCGATCTAAGTAAGCTTGGCATTCCGGTACT
GTTGGTAAAGCCACCATGGTGGCCGGGACCCGCTGTCTTCTAGCGTTGCTGCTTCCCCAGGTCCTC
CTGGGCGGCGCGGCTGGCCTCGTTCCGGAGCTGGGCCGCAGGAAGTTCGCGGCGGCGTCGTCGGG
CCGCCCCTCATCCCAGCCCTCTGACGAGGTCCTGAGCGAGTTCGAGTTGCGGCTGCTCAGCATGTT
CGGCCTGAAACAGAGACCCACCCCCAGCAGGGACGCCGTGGTGCCCCCCTACATGCTAGACCTGT
ATCGCAGGCACTCAGGTCAGCCGGGCTCACCCGCCCCAGACCACCGGTTGGAGAGGGCAGCCAGC
CGAGCCAACACTGTGCGCAGCTTCCACCATGAAGAATCTTTGGAAGAACTACCAGAAACGAGTGG
GAAAACAACCCGGAGATTCTTCTTTAATTTAAGTTCTATCCCCACGGAGGAGTTTATCACCTCAGC
AGAGCTTCAGGTTTTCCGAGAACAGATGCAAGATGCTTTAGGAAACAATAGCAGTTTCCATCACCG
AATTAATATTTATGAAATCATAAAACCTGCAACAGCCAACTCGAAATTCCCCGTGACCAGACTTTT
GGACACCAGGTTGGTGAATCAGAATGCAAGCAGGTGGGAAAGTTTTGATGTCACCCCCGCTGTGA
TGCGGTGGACTGCACAGGGACACGCCAACCATGGATTCGTGGTGGAAGTGGCCCACTTGGAGGAG
AAACAAGGTGTCTCCAAGAGACATGTTAGGATAAGCAGGTCTTTGCACCAAGATGAACACAGCTG
GTCACAGATAAGGCCATTGCTAGTAACTTTTGGCCATGATGGAAAAGGGCATCCTCTCCACAAAA
GAGAAAAACGTCAAGCCAAACACAAACAGCGGAAACGCCTTAAGTCCAGCTGTAAGAGACACCC
TTTGTACGTGGACTTCAGTGACGTGGGGTGGAATGACTGGATTGTGGCTCCCCCGGGGTATCACGC
CTTTTACTGCCACGGAGAATGCCCTTTTCCTCTGGCTGATCATCTGAACTCCACTAATCATGCCATT
GTTCAGACGTTGGTCAACTCTGTTAACTCTAAGATTCCTAAGGCATGCTGTGTCCCGACAGAACTC
AGTGCTATCTCGATGCTGTACCTTGACGAGAATGAAAAGGTTGTATTAAAGAACTATCAGGACATG
GTTGTGGAGGGTTGTGGGTGTCGCTAGTTAATTAATCTCGACGGTATCGGTTAACTTTTAAAAGAA
AAGGGGGGATTGGGGGGTACAGTGCAGGGGAAAGAATAGTAGACATAATAGCAACAGACATACA
AACTAAAGAATTACAAAAACAAATTACAAAAATTCAAAATTTTATCCATCACGAGACTAGCCTCG
AGGTTTAAAC

miR30a-shLuc

GTTTAAACCTAGAATATTGCTGTTTGAATGAGGCTTCAGTACTTTACAGAATCGTTGCCTGCACATC
TTGGAAACACTTGCTGGGATTACTTCTTCAGGTTAACCCAACAGAAGGCTAAAGAAGGTATATTGC
TGTTGACAGTGAGCGCCCGCCTGAAGTCTCTGATTAATAGTGAAGCCACAGATGTATTAATCAGAG
ACTTCAGGCGGTTGCCTACTGCCTCGGACTTCAAGGGGCTACTTTAGGAGCAATTATCTTGTTTACT
AAAACTGAATACCTTGCTATCTCTTTGATACATTTTTACAAAGCTGAATTAAAATGGTATAAATTA
AATCACTTTAAAACCATGGTTTAAAC

miR30a-shLHX8

GTTTAAACCTAGAATATTGCTGTTTGAATGAGGCTTCAGTACTTTACAGAATCGTTGCCTGCACATC
TTGGAAACACTTGCTGGGATTACTTCTTCAGGTTAACCCAACAGAAGGCTAAAGAAGGTATATTGC
TGTTGACAGTGAGCGCCCAACTGGCAGAAAGGACAGGCTAGTGAAGCCACAGATGTAGCCTGTCC
TTTCTGCCAGTTGGTTGCCTACTGCCTCGGACTTCAAGGGGCTACTTTAGGAGCAATTATCTTGTTT
ACTAAAACTGAATACCTTGCTATCTCTTTGATACATTTTTACAAAGCTGAATTAAAATGGTATAAA
TTAAATCACTTTAAAACCATGGTTTAAAC

Mouse Lhx8 CDS
ATGTATTGGAAGAGCGATCAGATGTTTGTGTGTAAGCTGGAGGGAAAGGAAGTGCCGGAGCTGGC
GGTTCCCCGCGAGAAGTGCCCCGGGCTCATGTCGGAGGAGTGCGGGCGGCCTGCAGCCCTGGCGG
CCGGGAGGACCCGCAAAGGCGCTGGGGAAGAAGGACTGGTGAATCCCGAGGGAGCCGGGGACGA
GGACTCCTGCTCCTCCTCGGCCCCGCTTTCCCCGTCGTCCTCGCCCCAGTCCATGGCCTCGGGCTCC
GTGTGCCCGCCAGGCAAGTGTGTGTGCAGCAGCTGCGGCCTGGAGATTGTGGACAAATACCTCCTC
AAGGTGAATGACTTATGCTGGCATGTCCGCTGTCTCTCCTGCAGTGTCTGCAGAACGTCCCTGGGA
AGGCACACGAGCTGCTACATTAAGGATAAAGACATTTTCTGTAAACTCGATTACTTCAGACGGTAT
GGGACCCGCTGTTCCCGCTGTGGCAGGCACATCCACTCGACTGACTGGGTCCGCAGGGCCAAGGG
CAACGTGTATCACCTGGCCTGCTTTGCCTGCTTTTCTTGCAAGAGGCAGCTGTCCACGGGAGAGGA
GTTCGCCTTGGTGGAGGAGAAGGTCCTCTGTAGAGTGCACTTTGACTGCATGCTGGACAATCTGAA
GAGAGAAGTGGAGAACGGTAATGGGATTAGTGTGGAAGGAGCCCTTCTCACAGAGCAAGACGTC
AATCATCCAAAGCCAGCCAAAAGAGCTCGGACCAGCTTCACAGCCGATCAGCTCCAGGTTATGCA
AGCACAGTTCGCTCAGGACAACAACCCAGATGCACAGACCCTCCAGAAACTGGCAGAAAGGACA
GGCTTAAGCAGACGTGTCATACAGGTGTGGTTTCAGAACTGCAGGGCCCGCCATAAGAAACACGT
CAGTCCCAACCATTCTTCCTCAGCCCCCGTCACAGCTGTCCCCTCCTCCAGGCTGTCCCCACCCATG
TTGGAAGAAATGGCTTATTCTGCCTACGTACCCCAGGATGGGACGATGCTGACTGCGCTGCACTCA
TACATGGATGCTCACCAACAACTCCTGGACTCCAGCCCTTGTTACCCCATCCAATGA

Supplementary Table 1 Primers used in the study.

Name	Sense	Antisense
Gapdh	5’-TGCCCTCATGTTCCTGATAAAT-3’	5’-CATTACATCACAGCTTTCCAGG-3’
Lhx8	5’-GGTGAATGACTTATGCTGGCATGT-3’	5’- CCATACCGTCTGAAGTAATCG -3’
Bmp2	5’-AGCAGGTCTTTGCACCAAGATGA-3’	5’- GCTGTTTGTGTTTGGCTTGACG -3’

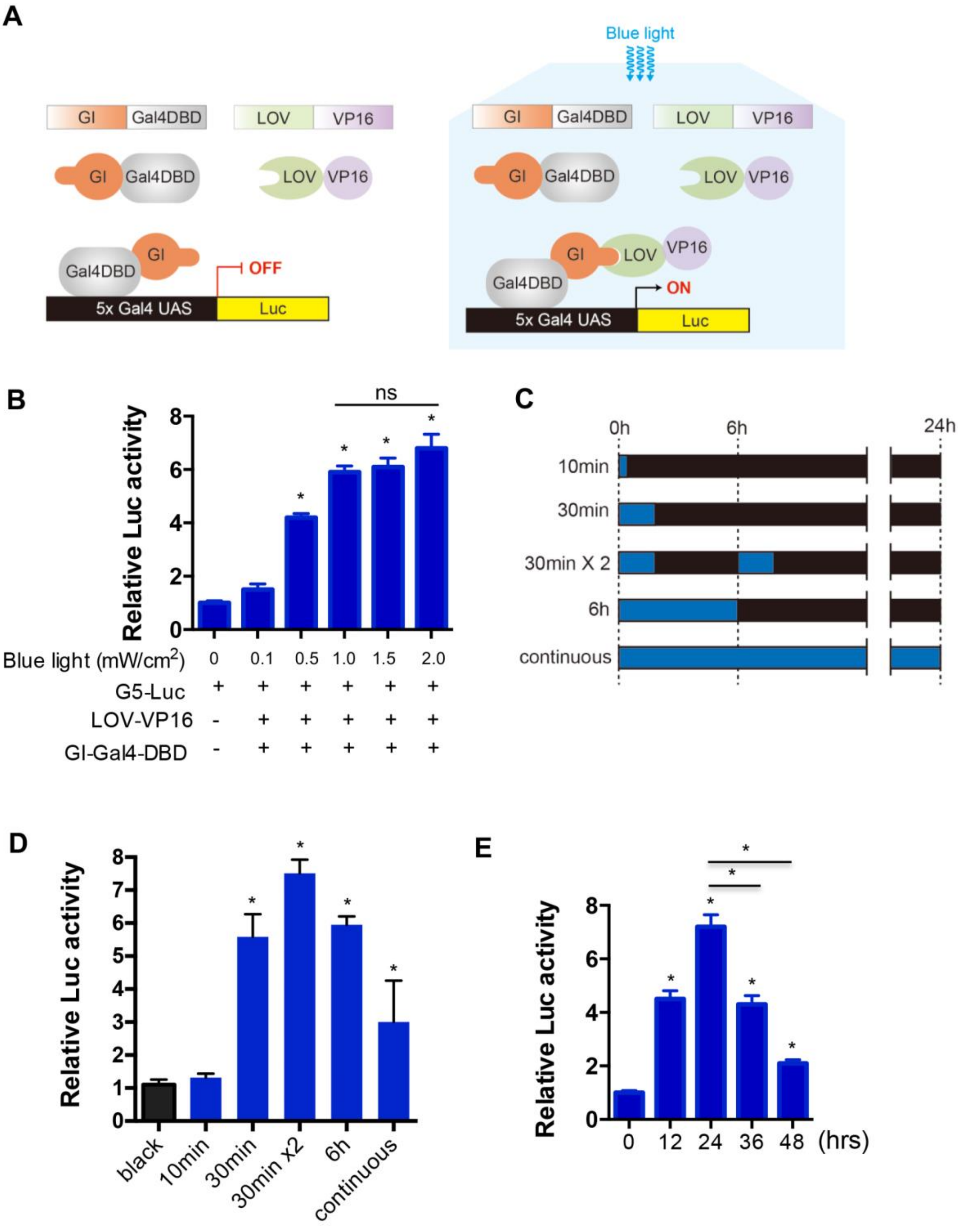

Supplementary Figure 1 Optimization of the light parameters to control gene expression. (A) Schematic representation of the protein structure of GI-Gal4DBD and LOV-VP16, and light induced interaction for transactivation of the luciferase under 5×Gal4 UAS promoter. (B) Relative luciferase activity of cells treated with different doses of blue light. HEK293 cells were co-transfected with GI-Gal4DBD, LOV-VP16, pG5luc and pRL-TK. Twenty-four hours later, cells were treated with or without 30 min blue light (0-2 mW/cm^2) before

harvested for luciferase activity. (C-D) Effects of light illumination duration on gene expression. (C) Schematic representation of the procedure of blue light illumination. (D) The relative luciferase activity of cells treated with blue light (1 mW/cm^2) for different duration. (E) Reversibility of light illumination on gene expression. HEK293 cells were co-transfected with GI-Gal4DBD, LOV-VP16, pG5luc and pRL-TK, followed by 30 min blue light illumination 24 h (1 mW/cm^2) after transfection. Luciferase activity was monitored at the indicated time post illumination. Data were expressed as mean±SEM, *P <0.05. n=5.

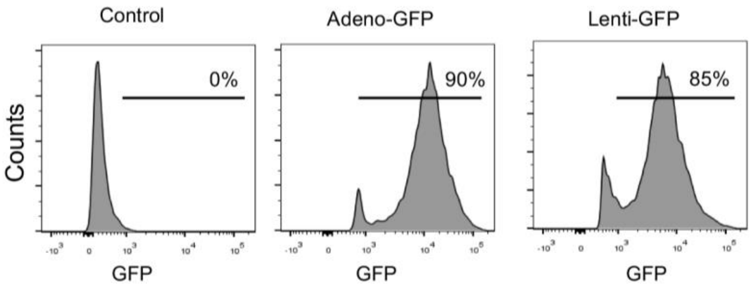

Supplementary Figure 2 Infection efficiency analyzed by flow cytometry. Control MSCs without virus infection or MSCs infected adnenovirus or lentivirus expressing GFP for 48 hours were subjected to flow cytometry for analysis of GFP expression. MSCs were efficiently infected by either adenovirus or lentivirus. Representative images of 3 different experiments.

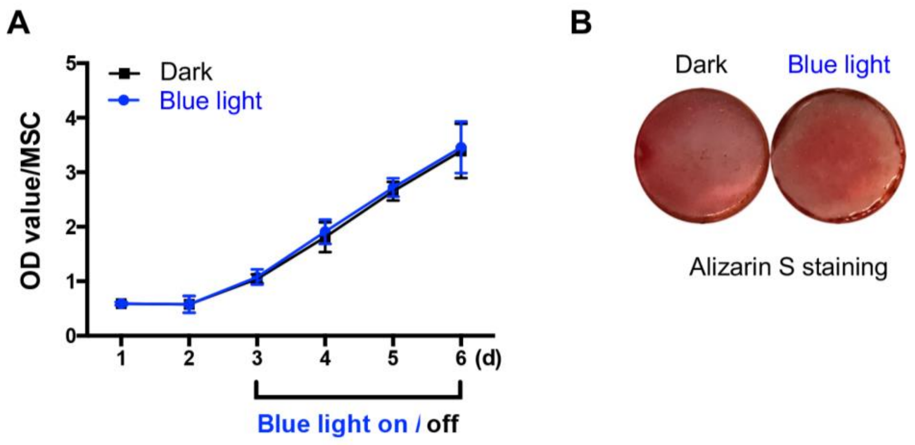

Supplementary Figure 3 Blue light alone has minimal effects on MSC fate in vitro. (A) Growth curve of the cells with or without blue light illumination. Primary MSCs without any gene transfection were cultured in dark until subjected to control (remaining dark) or blue light (30 min, 1 mW/cm^2) twice a day. Cell numbers were counted by CCK-8 assay. Data were expressed as mean±SEM, * P <0.05. n=5. (B) Representative images of the Alizarin red S staining of the cells treated similar as above, except that the cells were seeded and cultured in osteogenic medium.

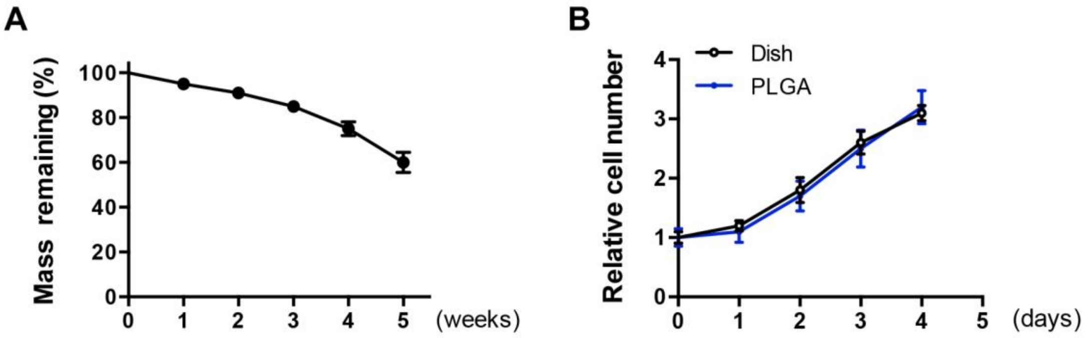

Supplementary Figure 4 Degradation of PLGA and its toxicity to MSCs. (A) Degradation rate of PLGA in PBS at RT. (B) CCK-8 analysis of cell growth in cell dish or PLGA sheets. Data were expressed as mean±SEM of three biological replicates.

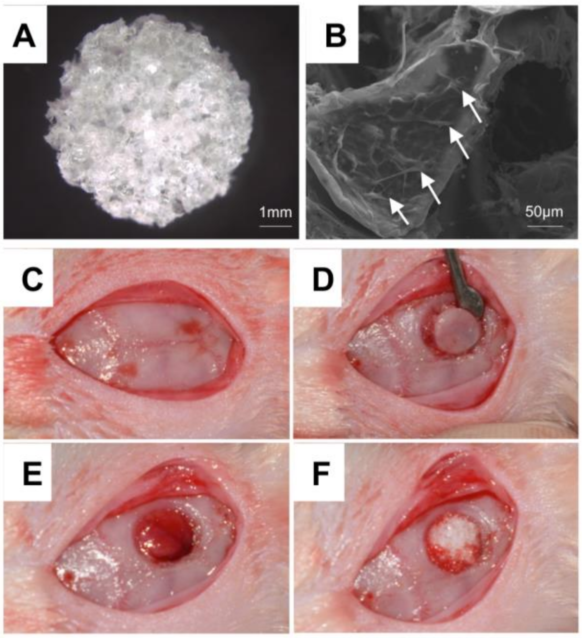

Supplementary Figure 5 Animal experimental procedures. (A) Morphology of the PLGA scaffold. (B) MSCs attached on the pores of the PLGA scaffold, as revealed by scanning electron microscope. (C-E) Surgery procedures showing the steps to produce calvarial defect (F) and MSCs/scaffold transplantation.

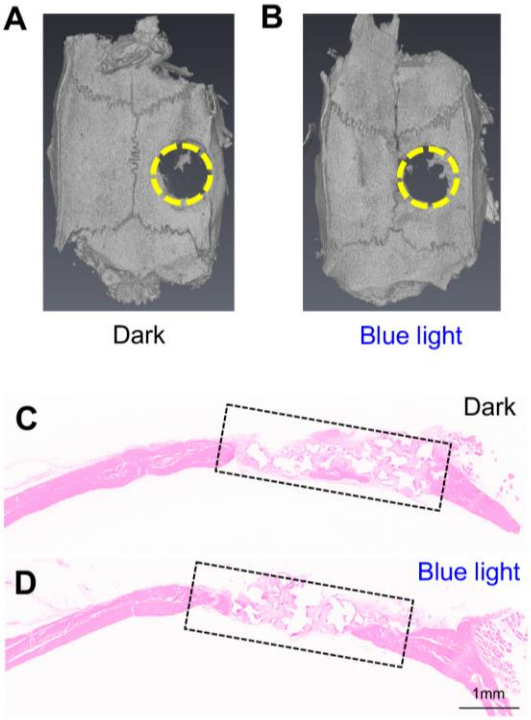

Supplementary Figure 6 Blue light alone has little effects on bone regeneration in vivo. Representative images from microCT (A-B) and HE staining (C-D) showing bone defect healing after 4 weeks of scaffold transplantation. The scaffold was loaded with MSCs without any transfection or infection. The rats were subjected to control (remaining in dark) or blue light illumination (30 min each time, twice a day) for 6 days.
